# Salidroside improves glucose homeostasis in obese mice by repressing inflammation in white adipose tissues and improving leptin sensitivity in hypothalamus

**DOI:** 10.1038/srep25399

**Published:** 2016-05-05

**Authors:** Meihong Wang, Lan Luo, Lili Yao, Caiping Wang, Ketao Jiang, Xiaoyu Liu, Muchen Xu, Ningmei Shen, Shaodong Guo, Cheng Sun, Yumin Yang

**Affiliations:** 1 Jiangsu Key Laboratory of Neuroregeneration, Nantong University, 19 Qixiu Road, Nantong, Jiangsu 226001, PRC; 2 Department of Geratology, Affiliated Hospital of Nantong University, Nantong, Jiangsu 226001, PRC; 3 Department of Endocrinology, Affiliated Hospital of Nantong University, Nantong, Jiangsu 226001, PRC; 4 School of Medicine, Nantong University, 19 Qixiu Road, Nantong, Jiangsu 226001, PRC; 5 Division of Molecular Cardiology, Department of Medicine, College of Medicine, Texas A&M University Health Science Center, USA

## Abstract

Salidroside is a functionally versatile natural compound from the perennial flowering plant *Rhodiola rosea* L. Here, we examined obese mice treated with salidroside at the dosage of 50 mg/kg/day for 48 days. Mice treated with salidroside showed slightly decreased food intake, body weight and hepatic triglyceride content. Importantly, salidroside treatment significantly improved glucose and insulin tolerance. It also increased insulin singling in both liver and epididymal white adipose tissue (eWAT). In addition, salidroside markedly ameliorated hyperglycemia in treated mice, which is likely due to the suppression of gluconeogenesis by salidroside as the protein levels of a gluconeogenic enzyme G6Pase and a co-activator PGC-1α were all markedly decreased. Further analysis revealed that adipogenesis in eWAT was significantly decreased in salidroside treated mice. The infiltration of macrophages in eWAT and the productions of pro-inflammatory cytokines were also markedly suppressed by salidroside. Furthermore, the leptin signal transduction in hypothalamus was improved by salidroside. Taken together, these euglycemic effects of salidroside may due to repression of adipogenesis and inflammation in eWAT and stimulation of leptin signal transduction in hypothalamus. Thus, salidroside might be used as an effective anti-diabetic agent.

Obesity and its related metabolic syndrome affect the health of >300 million people worldwide and are the third leading causes of preventable death^1^. Type 2 diabetes mellitus (T2DM) is a direct result from obesity, especially central obesity^2^. Numerous studies revealed that insulin resistance and hyperglycemia are the two hallmarks of T2DM^3^. Insulin resistance is a condition under which cells reduce or completely lose their capacity to stimulate glucose uptake responding to insulin^4^. To compensate glucose uptake, pancreatic β cells will produce more insulin and eventually β cell failure developed, which triggers the onset of T2DM^2^. In addition, it has been suggested that chronic and low-grade inflammation plays an important role in the development of obesity-induced insulin resistance^5^,^6^,^7^, and lowering inflammation has been proven to be an effective strategy for treating insulin resistance and T2DM^8^,^9^.

Currently, many pharmacological drugs are available for treating T2DM, such as metformin, sulfonylurea, alpha-glucosidase inhibitor and thiazolidinediones. While these drugs are able to effectively decrease the blood glucose level, they also possess side effects including hypoglycemia, weight gain and increased risk of heart failure^10^,^11^. To develop novel drugs, we focused on *R. rosea*, a medicinal plant from the Chinese traditional medicine that has been used as a potent tonic agent against altitude sickness in clinic^12^. Salidroside is a main active constituent of *R. rosea* and has demonstrated multiple functions such as anti-oxidative stress, anti-aging, anti-inflammatory and anti-fatigue, and protection of cells from exogenous insults induced by glutamate, heavy metal or ethanol^13^,^14^,^15^.

Our previous work showed that salidroside increases Akt phosphorylation in cultured rat hypothalamus^16^. Akt is a pivotal transducer and indicator in insulin signaling pathway^17^, we sought to investigate if salidroside may stimulate insulin signal pathway via enhanced Akt phosphorylation. To test the hypothesis that salidroside has an anti-diabetic effect, we administered salidroside to the mice fed with high fat diet (HFD) and the results showed that salidroside indeed improved glucose homeostasis in obese mice. This is likely due to decreased adipogenesis and inflammation in eWAT and increased leptin signal transduction in hypothalamus.

## Results

### Effects of salidroside on body weight and food intake

**Figure 1:**
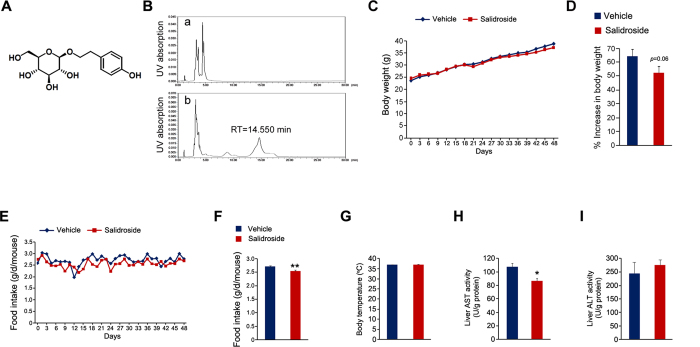
Salidroside administration reduces food intake in mice fed with high fat diet. (**A**) Chemical structure of salidroside. (**B**) The plasma salidroside concentrations were analyzed by HPLC. The representative elution profiles of plasma from vehicle treated (a) or salidroside treated mice (b). RT: retention time. (**C**) Body weight during the treatment. (**D**) The percent increase (%) in body weight. (**E-F**) Food intake. (**G**) Body temperatures. (**H**) AST activities in liver. (**I**) ALT activities in liver. All values represent mean ± SEM with n = 5 from three independent experiments. All data were analyzed by Student’s *t* test. *p < 0.05, **p < 0.01.

The schematic structure of salidroside is illustrated in Fig. 1A. To investigate its potential anti-diabetic effects, we administrated salidroside to the mice fed with HFD for 48 days. Briefly, salidroside was dissolved in distilled water at the concentration of 0.3 mg/ml and was freely accessed to the mice. Based on the average daily water consumption, the dosage of salidroside is around 50 mg/kg/day. To accurately determine the *in vivo* dosage of salidroside, we measured the plasma salidroside concentration by HPLC at the end of treatment. The results showed that the plasma salidroside concentration was 52.8 ± 9.4 μg/ml (~175 μM) (Fig. 1B), comparable to the dosage used in a cell model in which 240 μM of salidroside was found to be able to stimulate Akt phosphorylation^16^. We measured the body weight every 3 days throughout the treatment. As shown in Fig. 1C, the body weight was increased continuously in both the vehicle and salidroside treated animals. We calculated the percentage increase of body weight and found a mild decrease in the mice treated with salidroside (Fig. 1D). Food intake of the mice treated with salidroside was significantly lower than that of the control mice (Fig. 1E,F). No significant difference in body temperature was observed between the two groups (Fig. 1G). To exclude the possibility that salidroside may induce the liver toxicity and lead to a decrease in food intake, we measured aspartate transaminase (AST) and alanine transaminase (ALT) activities in liver. The results showed that the AST activity in liver was decreased by salidroside, whereas the ALT activity was not altered (Fig. 1H,I), indicating salidroside did not cause liver toxicity in our experiment. Our data showed that the application of salidroside induces a significant decrease in food intake and a mild reduction in body weight.

### Salidroside administration attenuates lipid accumulation in liver

**Figure 2:**
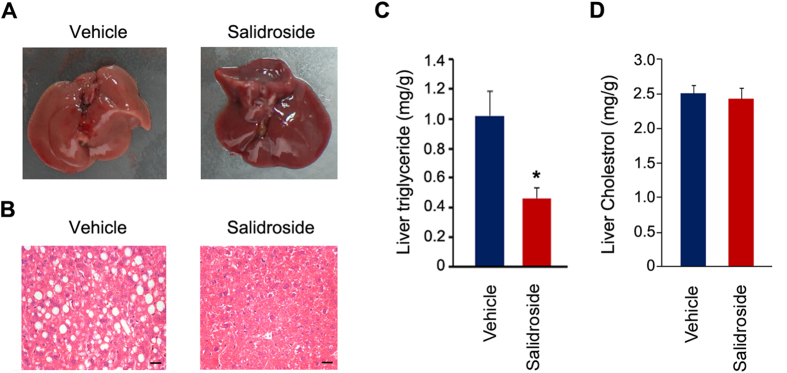
Salidroside administration attenuates lipid accumulation in livers of mice fed with high fat diets. (**A**) Liver gross morphology. (**B**) Liver sections by hematoxylin and eosin (H&E) staining. Scale bar represents 25 μm. (**C**) Analysis of liver triglyceride levels. (**D**) Analysis of liver total cholesterol levels. All values represent mean ± SEM with n = 5 from three independent experiments. Statistical significance was analyzed by Student’s *t* test. *p < 0.05.

At the end of the salidroside treatment, we found there was a striking difference in liver morphology between salidroside versus vehicle treated mice. As shown in Fig. 2A, the liver from HFD fed and vehicle treated mice was in a pale color, indicating the accumulation of hepatic lipids; whereas the liver of mice treated with salidroside was in a red-brown color, though the liver weight was not altered by salidroside treatment (data not shown). Based on the liver morphology, we predict that salidroside may reduce lipid accumulation in the liver. Indeed, the H&E staining results showed that the lipid droplets in vehicle treated mouse livers were much larger. In contrast, a few lipid droplets were seen in the livers of mice treated with salidroside (Fig. 2B). The triglyceride content in liver was markedly decreased (Fig. 2C). The cholesterol in liver was not altered by salidroside (Fig. 2D). These data indicate that salidroside treatment prevents lipid accumulation in livers of mice fed with HFD.

### Salidroside administration improves glucose and insulin tolerance and insulin signal transduction

**Figure 3:**
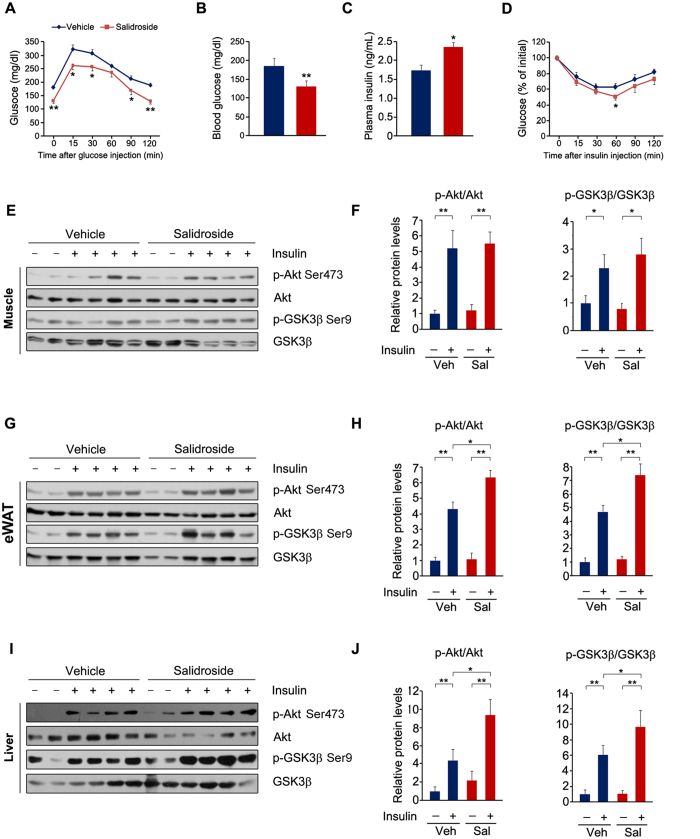
Salidroside administration improves glucose homeostasis and improves insulin signal transduction in livers of the obese mice. (**A**) Glucose tolerance test (GTT). (**B**) Plasma glucose levels. (**C**) Plasma insulin levels. (**D**) Insulin tolerance test (ITT). (**E**) Insulin signal transduction in the skeletal muscles. (**F**) Densitometric quantification of the immunoblot data in (**E**). (**G**) Insulin signal transduction in the white adipose tissues (WAT). (**H**) Densitometric quantification of the immunoblot data in (**G**). (**I**) Insulin signal transduction in the livers. (**J**) Densitometric quantification of the immunoblot data in (**I**). The protein levels of p-Akt Ser473 and p-GSK3β Ser9 were normalized to Akt and GSK3β protein levels, respectively. n = 6. All values represent mean ± SEM from three independent experiments. Data in (**B**,**C**) were analyzed by Student’s *t* test. Data in (**A**,**D**,**F**,**H**,**J**) compared by two-way ANOVA analysis. *p < 0.05, **p < 0.01.

Next we examined whether salidroside administration improves glucose and insulin tolerance in these obese mice. First, we performed glucose tolerance test and found salidroside markedly increased the glucose clearance rate (Fig. 3A). The fasting blood glucose levels were decreased and the plasma insulin levels were increased after salidroside treatment (Fig. 3B,C). Furthermore, the insulin tolerance was improved by salidroside (Fig. 3D). The improved glucose and insulin tolerance raised a question that whether insulin signal transduction was enhanced by salidroside. To test this, we measured insulin signaling in skeletal muscle, eWAT and liver after insulin stimulation. The results showed that the insulin-stimulated p-Akt and p-GSK3β were not altered by salidroside in the skeletal muscle (Fig. 3E,F). However, in the liver and eWAT, p-Akt and p-GSK3β levels were significantly increased in salidroside treated mice compared to vehicle treated mice (Fig. 3G–J). These data indicated that salidroside improves glucose homeostasis in HFD induced obese mice, which is likely due to enhanced insulin signal transduction in the liver and eWAT by salidroside.

### Salidroside represses hepatic gluconeogenesis

**Figure 4:**
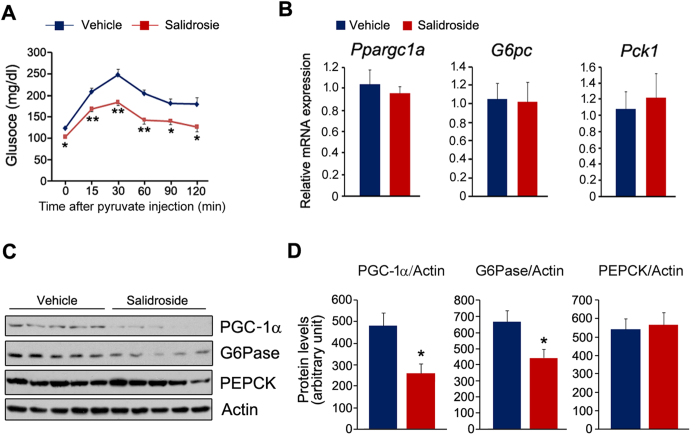
Salidroside treatment represses hepatic gluconeogenesis. (**A**) Pyruvate tolerance test (PTT). (**B**) The expression of *Ppargc1a*, *G6pc* and *Pck1*. (**C**) The protein levels of PGC-1α, PEPCK and G6Pase. (**D**) Densitometric quantification of the immunoblot data in (**C**). The protein levels of PGC-1α, PEPCK and G6Pase were normalized to Actin. n = 5. All values represent mean ± SEM from three independent experiments. Data in (**A**) compared by two-way ANOVA analysis. Data in (**D**) were analyzed by Student’s *t* test. *p < 0.05, **p < 0.01.

As salidroside enhances insulin signaling in the liver and increased hepatic gluconeogenesis impairs systemic glucose homeostasis, thus we investigated whether salidroside treatment affects gluconeogenesis in liver by using pyruvate tolerance test (PTT). As shown in Fig. 4A, salidroside remarkably repressed glucose synthesis from pyruvate. We next measured expressions of key genes in hepatic gluconeogenesis. The primer sequences were listed in Table 1. Though the mRNA levels of *Ppargc1a*, *Pck1* and *G6pc* were not altered by salidroside (Fig. 4B), the protein levels of PGC-1α and G6Pase were significantly decreased, whereas PEPCK was not affected (Fig. 4C,D). These data showed that salidroside represses hepatic gluconeogenesis possibly by decreasing PGC-1α and G6Pase expressions at the protein level.

### Salidroside has no effects on AMPK and UPR signaling pathways

**Figure 5:**
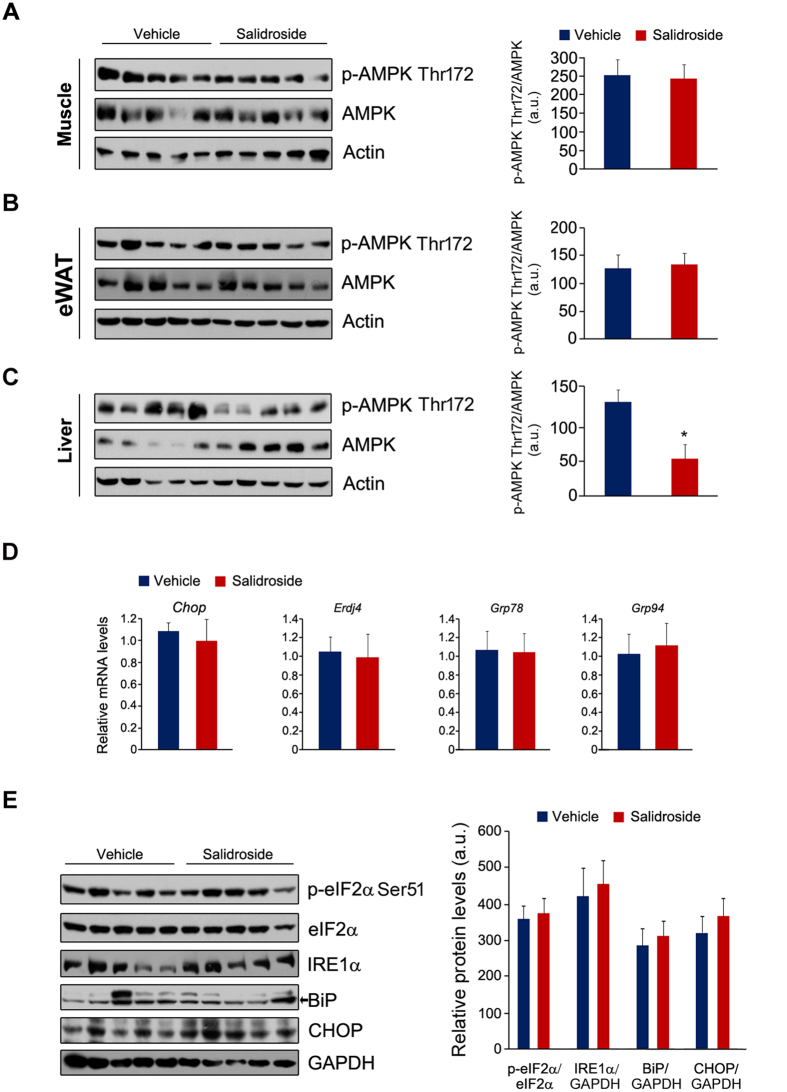
AMPK and unfold protein response (UPR) pathways were not affected by salidroside. (**A**–**C**) The protein levels of p-AMPK and AMPK in the skeletal muscle (**A**), eWAT (**B**) and liver (**C**). (**D**) The chaperon gene expressions in the liver. (**E**) The protein levels of p-eIF2α, eIF2α, IRE1α, BiP and CHOP in the liver. GAPDH was used as loading control. The p-eIF2α levels were normalized to eIF2α. The protein levels of IRE1α, BiP and CHOP were normalized to GAPDH. eWAT: epididymal white adipose tissue. a.u. means arbitrary unit. n = 5. All values represent mean ± SEM from three independent experiments. All data were analyzed by Student’s *t* test. *p < 0.05.

AMPK is a promising target for improving the insulin signaling transduction^18^. A recent study demonstrated that salidroside treatment attenuates insulin resistance in db/db mice by stimulating AMPK^19^. To confirm this conclusion, we measured AMPK in muscle, eWAT and liver. However, the phospho-AMPK (p-AMPK) and AMPK were not affected by salidroside in skeletal muscle and eWAT (Fig. 5A,B). In liver, the p-AMPK was even decreased (Fig. 5C). Increased endoplasmic reticulum stress (ER stress) in liver was also reported as a pivotal factor for inducing insulin resistance^20^. Therefore, we examined whether ER stress in liver was altered by salidroside. The chaperon gene expressions were not affected by salidroside (Fig. 5D). Similarly, the protein levels of p-eIF2α, eIF2α, IRE1α, BiP and CHOP were the same between the control mice and the salidroside treated animals (Fig. 5E). These data show that AMPK and ER stress in liver were not the mechanisms by which salidroside improves glucose and insulin tolerance in HFD induced obese mice.

### Salidroside represses adipogenesis in epididymal white adipose tissues

**Figure 6:**
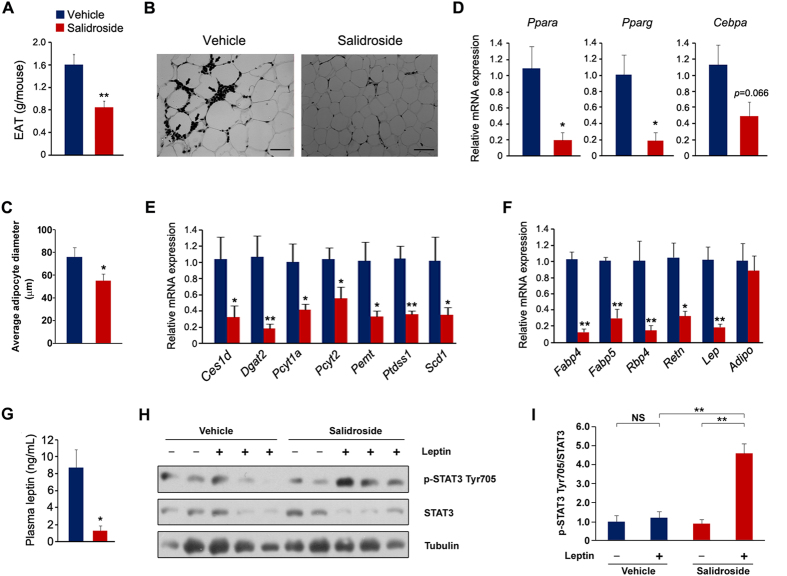
The application of salidroside reduces adipogenesis in white adipose tissues. (**A**) Epididymal white adipose tissue (eWAT) weight. (**B**) Epididymal adipose tissue sections by hematoxylin and eosin (H&E) staining. Arrows indicate macrophage-like cells. Bar represents 50 μm. (**C**) Average diameter of adipocytes of eWAT. Adipocyte sizes were quantified from 100 adipocytes from 10 fields. (**D**) The gene expressions of key transcriptional factors in adipogenesis. (**E**) The expressions of genes involved in triglyceride synthesis. (**F**) The adipocytokine gene expressions. (**G**) The plasma leptin levels. (**H**) Leptin signaling pathway in hypothalamus. (**I**) Densitometric quantification of the immunoblot data in (**H**). The p-STAT3 Tyr705 protein levels were normalized to total STAT3 protein levels. n = 5. All values represent mean ± SEM from three independent experiments. All data except in (**I**) compared by Student’s *t* test. Data in (**I**) were analyzed by two-way ANOVA analysis. *p < 0.05, **p < 0.01.

In addition to the difference in liver morphology, we also observed the smaller size of eWAT in salidroside treated mice. As shown in Fig. 6A, eWAT weight was decreased remarkably by salidroside. The H&E staining revealed that the adipocytes of eWAT from the salidroside-treated animals were smaller (Fig. 6B,C). These results prompt us to investigate whether adipogenesis is affected by salidroside. To test this, we measured the expressions of several key transcriptional factors in adipogenesis. The mRNA levels of *Ppara* and *Pparg* were significantly reduced by salidroside (Fig. 6D). The mRNA level of *Cebpa* was also decreased, although the reduction did not reach statistical significance (Fig. 6D). The gene expressions involved in adipogenesis were largely decreased by salidroside (Fig. 6E). Moreover, we also examined gene expressions of adipocytokines. As shown in Fig. 6F, the mRNA levels of *Fabp4*, *Fabp5*, *Rbp4, Retn* and *Lep* were markedly reduced, whereas *Adipo* was unaffected. Additionally, we measured the plasma leptin levels and the results showed that there is a sharp decline in the salidroside-treated animals (Fig. 6G). To examine whether leptin signal transduction was improved by salidroside, we measured phospho-STAT3 (p-STAT3) levels in hypothalamus. The results showed that p-STAT3 levels were not affected by the administration of leptin in the vehicle-treated animals. However, p-STAT3 levels were markedly stimulated by leptin in the salidroside-treated mice (Fig. 6H,I). These results clearly indicate that salidroside treatment suppresses adipogenesis in eWAT and improves leptin signaling transduction in hypothalamus.

### Salidroside reduces inflammation in epididymal white adipose tissues

**Figure 7:**
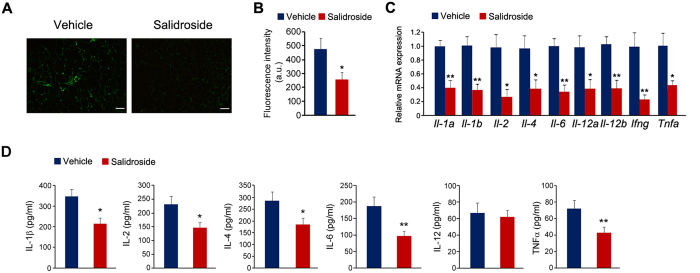
The application of salidroside decreases inflammation in white adipose tissues. (**A**) Immunostaining of macrophages in eWAT by anti-CD68 antibody. Scale bar represents 100 μm. (**B**) Fluorescence intensity of CD68-positive cells in eWAT. a.u. means arbitrary unit. (**C**) The expressions of inflammatory cytokines. (**D**) The plasma levels of IL-1β, IL-2, IL-4, IL-6, IL-12 and TNFα. n = 5. All values represent mean ± SEM from three independent experiments. All data were analyzed by Student’s *t* test. *p < 0.05, **p < 0.01.

Consistent with well documented macrophage infiltration in WAT in obese mice^21^, we observed macrophage infiltration in the interval space of adipocytes when mice were fed with the HFD (Fig. 6B, arrow). Increased macrophage infiltration is often associated with increased production of inflammatory cytokines. Therefore we examined whether salidroside represses macrophage infiltration and reduces the productions of inflammatory cytokines derived from macrophages. To test this, we performed immunostaining using antibody against a macrophage marker CD68. Our results showed that a lot of CD68-positive cells were observed in the eWAT of the vehicle treated mice, but just a few CD68-positive cells were observed in the salidroside treated mice (Fig. 7A). The fluorescence intensity of CD68-positive cells in eWAT from the vehicle treated mice was significantly higher than that from the salidroside treated animals (Fig. 7B). Furthermore, we evaluated the inflammatory cytokine expressions. The data showed that the expressions of *Il*-*1a*, *Il*-*1b*, *Il*-*2*, *Il*-*4*, *Il*-*6*, *Il*-*12*, *Ifng* and *Tnfa* were dramatically decreased by salidroside (Fig. 7C). To further confirm these results, we also measured the plasma levels of several inflammatory cytokines. As shown in Fig. 7D, the plasma levels of IL-1β, IL-2, IL-4, IL-6 and TNFα were significantly decreased by salidroside. These data indicate that the application of salidroside greatly attenuates inflammation in WAT of obese mice.

### Salidroside has minimal euglycemic effects in ob/ob mice

**Figure 8:**
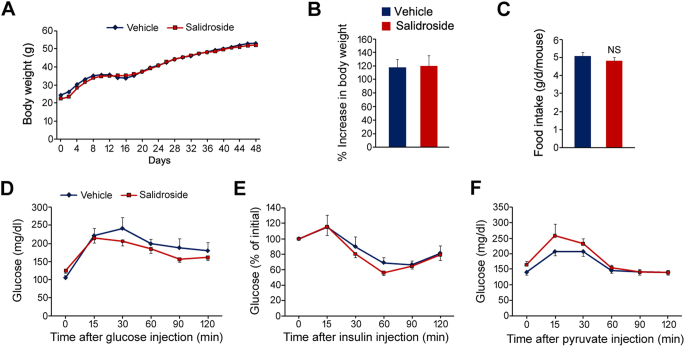
Salidroside has no euglycemic effects in ob/ob mice. (**A**) Body weight during the treatment. (**B**) The percent increase (%) of body weight. (**C**) Food intake. (**D**) Glucose tolerance test (GTT). (**E**) Insulin tolerance test (ITT). (**F**) Pyruvate tolerance test (PTT). All values represent mean ± SEM from two independent experiments. Data in (**B**,**C**) were analyzed by Student’s *t* test. Data in (**D**–**F**) were analyzed by two-way ANOVA assay. n.s. means no significance.

To uncover the precise mechanisms for the salidroside mediated euglycemic effects in HFD-induced obese mice, we treated leptin deficient (ob/ob) mice with salidroside by the same strategy. The results showed that the body weight of ob/ob mice was not affected by salidroside (Fig. 8A,B). The food intake was not altered either (Fig. 8C). The glucose tolerance, as well as insulin tolerance, was unaltered by salidroside in ob/ob mice (Fig. 8D,E). The hepatic gluconeogenesis was even stimulated by salidroside to some extent (Fig. 8F). These data indicated that salidroside-mediated euglycemic effects in obese mice mainly depend upon the activation of leptin signal transduction in hypothalamus.

## Discussion

In this study, we demonstrated that: 1) salidroside at a dosage of 50 mg/kg/day improved glucose homeostasis in obese and insulin resistant mice induced by the high fat diet; 2) salidroside administration ameliorates fatty liver in these mice; 3) salidroside treatment reduces adipogenesis in epididymal adipose tissue and down-regulates several pro-inflammatory cytokine productions; 4) the euglycemic effects of salidroside mainly depend upon activation of leptin signal transduction in hypothalamus.

Insulin resistance or impaired insulin signaling is a main cause for malfunctioned glucose metabolism and plays a pivotal role for the pathogenesis of T2DM^4^. Reciprocally, improvement or restoration of insulin signaling is an effective strategy for rectifying abnormal glucose metabolism in T2DM. In the insulin signaling pathway, Akt is a central mediator for insulin signaling transduction and numerous evidences revealed that chemicals stimulating Akt have potential efficacies for improving insulin signal transduction^17^,^22^,^23^,^24^. Our previous work demonstrated that MADP, a salidroside derivative, robustly activates Akt in the cultured hypothalamic neurons^16^. In addition to MADP, salidroside itself also possesses the ability to activate Akt^16^. It is reasonable to predict that salidroside may maintain euglycemia by improving the insulin signaling pathway in type 2 diabetic mice. To test this hypothesis, we first treated HepG2 hepatocytes and C2C12 myotubes with salidroside to examine whether salidroside stimulates insulin signal transduction in these cells. However, the insulin signal transduction was not affected by salidroside in HepG2 and C2C12 cells (data not shown). On the contrary, the Akt and GSK3β phosphorylation in liver and eWAT were strengthened by salidroside. Moreover, the administration of salidroside really improves glucose homeostasis in the high fat diet induced obese mice. These data indicate that salidroside may play an indirect effect on insulin signal transduction in obese mice.

The reasons for the improved insulin signal transduction in liver and eWAT are probably due to the reductions in circulated pro-inflammatory cytokines. The inflammation in white adipose tissues is widely considered as a causative factor for inducing systemic insulin resistance^4^. Pro-inflammatory cytokines, such as TNFα, IL-1β and IL-6, impair the insulin sensitivity of local adipocytes as well as that of liver and muscle^6^. Here, we have shown that the expressions of pro-inflammatory cytokines in white adipose tissues are greatly suppressed by salidroside. In agreement with our results, it has been shown that salidroside inhibits the productions of IL-6, IL-1β and TNFα in the gastric epithelial cells treated with H_2_O_2_^15^. In addition to pro-inflammatory cytokines, other cytokines secreted from adipose tissues also play an important role for the insulin signaling pathway. For example, resistin and Rbp4 synthesized in white adipose tissues block insulin signal transduction and cause insulin resistance^25^,^26^. FABP4 is another adipocytokine which disturbs glucose metabolism by stimulating glucose production and gluconeogenic activity^27^,^28^. These gene expressions were markedly decreased by salidroside. The other reason for the improved insulin signal transduction may result from the enhanced plasma insulin levels. The underlying molecular mechanism for salidroside-induced up-regulation of insulin remains unclear. We predict that salidroside might stimulate insulin synthesis and/or secretion in pancreatic β cells. Alternatively, salidroside might protect pancreatic β cells from apoptosis in type 2 diabetic mice.

In addition to the inflammation in eWAT, the adipose mass was also affected by salidroside. Our results revealed a remarkable reduction in eWAT in the mice treated with salidroside. The gene expression analysis showed that the key transcriptional factor, PPARα and PPARγ expressions are down-regulated by salidroside. This is consistent with previous studies that have shown repression effects of salidroside on adipogenesis in *in vitro* models^29^,^30^. Notably, PPARγ is a master transcriptional factor for adipogenesis in WAT. Down-regulation of PPARγ in adipocytes decreases fat mass and PPARγ knockout mice completely lack adipose tissue^31^,^32^. In the current study, we observed that PPARγ in eWAT was greatly down-regulated by salidroside. Accumulating evidence has shown that excessive fat mass is a contributing factor to pathophysiology of the metabolic derangements in obesity and T2DM^33^. In this regard, suppression of adipogenesis and fat mass is an effective strategy for improving glucose metabolism in obese animals. Numerous studies have proven this notion^34^,^35^,^36^. Herein, we also showed that salidroside-induced fat mass reduction associates with the improved glucose homeostasis in HFD induced obese mice.

48-day treatment with salidroside led to a small, though not statistical significant, decrease in body weight of the mice fed with HFD. The food intake was significantly repressed by salidroside, a reason likely to explain the decreased body weight. No toxicity in liver was occurred in the animals treated with salidroside at the dosage of 50 mg/kg/day. Thus, the decrease in body weight likely is directly resulted from the decline in food intake. Leptin, secreted from the WAT, acts in hypothalamus and initiates a cascade signaling transduction to suppress appetite^37^. As a result, leptin induces the expressions of pro-opiomelanocortin (POMC) and cocaine- and amphet-amine-regulated transcript (CART), which cause a decrease in food intake^37^. Plasma leptin levels were increased markedly in obese animals as a consequence of leptin resistance^38^. In the present study, we observed the leptin sensitivity in hypothalamus was improved by salidroside. In accordance to our findings, it has been reported recently that, celastrol, a natural compound from the roots of thunder god vine, exhibits great anti-obesity efficacy in diet induced obese mice by improving leptin sensitivity in hypothalamus^35^. However, in leptin deficient (ob/ob) mice, salidroside application plays minimal euglycemic effects. These data clearly indicate that the salidroside-mediated euglycemic effects in HFD induced obese mice mainly depend on activation of leptin sensitivity in hypothalamus. Other reports proposed that the salidroside plays a hypoglycemic role in diabetic mice via AMPK activation or anti-oxidative stress pathway^19^,^39^. These discrepancies may be derived from variances in experimental animal species and/or salidrosdie dosages and/or treating regimens.

Gluconeogenesis is a metabolic pathway in which glucose is generated from non-carbon substrates such as pyruvate, lactate, glycerol and glucogenic amino acids. Ectopic up-regulation of gluconeogenesis in the liver is a cause for hyperglycemia, whereas repression of gluconeogenesis is an effective strategy for lowering glucose in diabetic animals^40^,^41^. Our results showed that salidroside markedly reduces glucose production after pyruvate injection, indicating the hepatic gluconeogenesis is repressed by salidroside. Consistent with our findings, *Rhodiola crenulata* extract could suppress hepatic gluconeogenesis in rat liver via AMPK activation^42^. Most recently, it has been shown that salidroside treatment attenuates the hepatic gluconeogenesis in db/db mice by down-regulating PEPCK and G6Pase^19^. The salidroside-mediated repression on hepatic gluconeogenesis may be due to the increased insulin levels in salidroside-treated mice, since insulin is a potent inhibitor of gluconeogenesis. PGC-1α is the key co-activator to stimulate hepatic gluconeogenesis pathway by inducing the expressions of *G6pc* and *Pck1,* both of which are the rate-limited enzymes in gluconeogenesis^43^. Herein, we found salidroside treatment leads to a decrease in the protein levels of PGC-1α and G6Pase in liver. However, salidroside had no effect on their mRNA levels, indicating that salidroside may interferes with the translational processes of PGC-1α and G6Pase, or accelerate the degradation of these proteins.

In a previous study, Zheng and colleagues proposed that the activation of AMPK induced by salidroside is the main underlying mechanism for the improved insulin signaling pathway in db/db mice^19^. Another report found that salidroside improves glucose uptake in L6 myotubes by activating AMPK^44^. In the present work, we also detected AMPK in the muscle, eWAT and liver from HFD induced obese mice. However, the AMPK was not altered by salidroside in skeletal muscle and WAT. In liver, AMPK was even decreased by salidroside. Moreover, we found that salidroside application has minimal euglycemic effects in leptin deficient (ob/ob) mice. On the contrary, in leptin receptor deficient (db/db) mice, salidroside treatment ameliorates insulin resistance and thus plays beneficial roles against diabetes^19^. These discrepancies may result from differences in mouse models (db/db mice versus HFD induced obese mice or ob/ob mice).

Taken together, our results showed that salidroside, a natural product of *R. rosea*, possesses euglycemic effects in the high fat diet induced obese mice. These euglycemic effects likely depend upon the salidroside induced suppression on food intake and body weight gain, which eventually leads to reductions in adipogenesis and inflammation in eWAT. Therefore, salidroside might be used as a potential therapy for treating obesity-related metabolic diseases such as insulin resistance and hyperglycemia.

## Methods

### Biochemical reagents

Salidroside (purity >98%) was from National Institute for Food and Drug Control (Beijing, China). The high fat diet (45% fat) was from Research Diets (New Brunswick, NJ). The antibodies anti-phospho-Akt Ser473, anti-Akt, anti-phospho-GSK3β Ser9, anti-GSK3β, anti-phospho-eIF2α Ser51, anti-eIF2α, anti-phospho-AMPK Thr172, anti-AMPK, anti-phopho-STAT3 Tyr705, anti-STAT3, anti-BiP, anti-PERCK, anti-IRE1α, anti-Tubulin and anti-CHOP were from Cell Signaling Technology (Beverley, MA). The antibodies anti-CD68, anti-PGC-1α and anti-G6Pase were purchased from Abcam (Cambridge, MA). The antibodies anti-Actin, anti-GAPDH, pyruvate, glucose, insulin, EGTA, EDTA, NP-40, leupeptin, aprotinin, PMSF and okadaic acid were from Sigma-Aldrich (St. Louis, MO). Recombinant mouse leptin was from R&D systems (Minneapolis, MN). cDNA Synthesis Kit, SYBR Green Supermix and Detergent-compatible protein assay kit were from Bio-Rad (Hercules, CA). Chemiluminescence blotting substrate was from Roche (Indianapolis, IN). The other chemical reagents were of analytical grade.

### Mice

6-week old C57BL/6J mice were fed with high fat diets (45% of calories from fat, Research Diets). Salidroside was dissolved in distilled water at 0.3 mg/ml and given to mice with free access. The mice given distilled water were used as control. Salidroside administration is initiated when the mice fed with high fat diets. 4-week old leptin deficient (ob/ob) mice were received salidroside as described above. At the 30th and 36th day, glucose tolerance test and insulin tolerance test were performed, respectively. At the 42th day, pyruvate tolerance test was carried out. At the 48th day, the mice were anesthetized and sacrificed after a 6 h-fasting. Blood was taken from eyehole using capillary tubes and placed on ice. Plasma were obtained after centrifugation at 12,000 rpm for 20 min and stored in −80 °C freezer until use. Gastrocnemius muscle, liver and epididymal fat tissue were removed and snap frozen in liquid nitrogen. For longer storage, tissues were placed in −80 °C freezer. All of the animal protocols were approved by the Animal Care and Use Committee of Nantong University and the Jiangsu Province Animal Care Ethics Committee. All the methods were carried out in accordance with the approved guidelines.

### Body weight, food intake and body temperature assays

For HFD induced obese mice, body weight and food intake was measured every 3 days. For ob/ob mice, body weight and food intake were monitored every other day. Core body temperature was measured rectally with a thermistor (Micro-Therma 2T/ThermoWorks) during the light cycle once a week.

### Plasma salidroside detection

Plasma (50 μl) was mixed with 200 μl of acetonitrile, vortexed for 5 min, and then centrifuged at 12,000 rpm for 10 min. The supernatant was evaporated to dryness under a stream of N_2_. The residue was reconstituted in 50 μl C_2_H_5_OH/H_2_O (20:80 v/v). After centrifugation at 12,000 rpm for 5 min, 10 μl of the resulting supernatant was subjected to HPLC analysis. The HPCL system (Waters 2695, Milford, USA) consisted of a Waters 2998 photodiode array detector, a vacuum degasser, an autosampler, a column thermostat, and a Waters Symmetry 300TM C18 column (250 × 4.6 mm id, 5.0 μm particle size). The column oven temperature was fixed at 30 °C. A mobile phase was consisted of methanol and water (10:90 v/v). The flow rate of mobile phase was set at 1.0 ml/min and the wavelength of UV detector was set at 278 nm.

### Blood glucose, plasma insulin and leptin assays

Mice were fasted for 6 h, after which their blood was analyzed for glucose measurement with a glucose meter (Bayer, Mishawaka, IN). For insulin analysis, mice were fasted for 6 h and plasma insulin were measured with an Ultra Sensitive Mouse Insulin ELISA kit from Crystal Chem (Downers Grove, IL). Plasma leptin levels were analyzed by an ELISA kit from Life technologies (Thermo Fisher Scientific).

### Liver ALT and AST activity assays

Liver ALT and AST activities were measured by kits (Jiancheng Bioengineering Institute, Nanjing, China) according to the manufacturer’s instructions.

### Triglyceride and cholesterol measurements

Triglyceride and cholesterol in liver were determined using kits from Sigma-Aldrich according to the manufacturer’s instructions.

### Glucose tolerance test (GTT), insulin tolerance test (ITT) and pyruvate tolerance test (PTT)

For GTT analysis, mice were intraperitoneally injected with D-glucose (0.5 g/kg) after an overnight fasting. For ITT analysis, mice were fasted for 6 h (from 8 am to 2 pm) and intraperitoneally injected with recombinant human insulin (0.75 IU/kg) from Eli Lilly (Indianapolis, IN). For PTT analysis, mice were fasted for 18 h and intraperitoneally injected with pyruvate (1 g/kg). Blood was taken from tail vein at 0, 15, 30, 60, 90 and 120 min after glucose or insulin or pyruvate injection and blood glucose was measured with a glucometer from Bayer.

### Tissue protein extraction

Tissues were homogenized with a bench-top homogenizer (Polytron, PT2100) in ice-cold tissue lysis buffer (25 mM Tris-HCl, pH 7.4; 100 mM NaF; 50 mM Na_4_P_2_O_7_; 10 mM Na_3_VO_4_; 10 mM EGTA; 10 mM EDTA; 1% NP-40; 10 μg/ml Leupeptin; 10 μg/ml Aprotinin; 2 mM PMSF and 20 nM Okadaic acid). After homogenization, lysates were rotated for 1 h at 4 °C and then subjected to centrifugation at 13,200 rpm for 20 min at 4 °C. The lipid layer was removed and the supernatant was transferred into Eppendorf tubes for centrifugation. This process was repeated for twice to get rid of lipid completely. Protein concentration was quantified by using Protein Assay Kit (Bio-Rad). Equivalent protein concentration in each sample was prepared and boiled at 100 °C for 5 min in Laemmli buffer. The lysates were cooled to room temperature before loading for Western blot analysis.

### Western blot analysis

Western blot analysis was performed as previously described^24^. Samples from cell lysates or tissue lysates were resolved by SDS-PAGE and then transferred to polyvinylidene fluoride (PVDF) membrane. After 1 h blocking at room temperature using 10% blocking reagent (Roche), membrane was incubated overnight with primary antibody in Tris-buffered saline solution/Tween (TBST) containing 10% blocking reagent at 4 °C. After the incubation, membrane was washed three times in TBST and incubated with secondary antibody for 1 h at room temperature. After three-time washing in TBST, membrane was developed using a chemiluminescence assay system (Roche) and exposed to Kodak exposure films. Relative protein levels were quantified by Image J program. For stripping, membrane was vigorously shaken in stripping buffer (62.5 mM Tris-HCl, pH 6.7; 2% SDS; 100 mM 2-mecaptomethanol) at 50 °C for 20 min. After stripping, membrane was washed three times in TBST and re-blotted with other antibodies with the procedures as described above.

### Analysis of *in vivo* insulin signaling

For *in vivo* insulin signaling analysis, mice were received insulin (0.75 IU/kg) via intraperitoneal injection after 6 h of fasting. Five minutes after injection, gastrocnemius muscle, liver and epididymal white adipose tissue were removed and snap frozen in liquid nitrogen and stored at −80 °C until use.

### Analysis of leptin signaling transduction

Mice were intraperitoneally injected with saline or leptin (1 mg/kg) and 30 min later, hypothalamuses were collected for analyzing phospho-STAT3 Tyr705 and total STAT3 by Western blot.

### Total RNA extraction and real-time quantitative PCR

Total RNA was extracted from cells or animal tissues using Trizol reagent (Invitrogen) and transcribed into cDNA using cDNA synthesis kit (Bio-Rad). The gene expression analysis was performed with iQ5 Multicolor Real-Time PCR Detection System (Bio-Rad) with SYBR Green Supermix (Bio-Rad). The comparative 2^−ΔCt^ method was applied to calculate mRNA levels. The mRNA level was normalized to a house keeping gene 18S. The primer sequences were listed in Table 1.

### Histochemistry and immunostaining

Paraffin sections of liver and adipose tissue were fixed in 4% paraformaldehyde and were stained with hematoxylin and eosin (H&E) for histology. For analyzing macrophages in adipose tissue, paraffin sections of adipose tissue were incubated with mouse anti-CD68 antibody. After washing, slides were incubated with FITC-conjugated goat anti-mouse IgG (Life Technologies). The CD68-positive cells were visualized by an Olympus FluoView FV 1000 microscope (Tokyo, Japan).

### Fluorescence intensity analysis

Fluorescence pixel intensities in CD68-positive cells in eWAT were measured by ImageJ as described previously^45^.

### Plasma pro-inflammatory cytokine determination

Plasma pro-inflammatory cytokine levels, such as IL-1β, IL-2, IL-4, IL-6, IL-12 and TNFα, were quantified by using ELISA kits (R&D) according to their manual instructions.

### Statistical analysis

Data are presented as means ± standard error of the mean (SEM). SPSS 18.0 was used for statistical analysis. Comparisons between two groups, paired Student’s *t* test, and comparison between two factors two-way ANOVA followed by post hoc testing (Bonferroni correction) were conducted. Statistical significance was set at p < 0.05 (*) and p < 0.01 (**).

## Additional Information

**How to cite this article**: Wang, M. *et al.* Salidroside improves glucose homeostasis in obese mice by repressing inflammation in white adipose tissues and improving leptin sensitivity in hypothalamus. *Sci. Rep.* **6**, 25399; doi: 10.1038/srep25399 (2016).

## Figures and Tables

**Table 1:** Primer sequences.

Gene	Forward primer	Reverse primer
18S	AGTCCCTGCCCTTTGTACACA	CGTTCCGAGGGCCTCACT
Erdj4	CCCCAGTGTCAAACTGTACCAG	AGCGTTTCCAATTTTCCATAAATT
Grp78	TCATCGGACGCACTTGGAA	CAACCACCTTGAATGGCAAGA
Grp94	TCGTCAGAGCTGATGATGAAGT	GCGTTTAACCCATCCAACTGAAT
Chop	CTGGAAGCCTGGTATGAGGAT	CAGGGTCAAGAGTAGTGAAGGT
G6pc	CCGGTGTTTGAACGTCATCT	CAATGCCTGACAAGACTCCA
Pck1	ATCATCTTTGGTGGCCGTAG	ATCTTGCCCTTGTGTTCTGC
Ppargc1a	TGATGTGAATGACTTGGATACAGACA	CAATGCCTGACAAGACTCCA
Ppara	TGATGTGAATGACTTGGATACAGACA	CAATGCCTGACAAGACTCCA
Pparg	CCTGATGAATAAAGATGGAGTC	AATATAGCCAAGTCACTGTCA
Cebpa	CAAACTGAGACTCTTCACTAAC	CTAAGACCCACTACTACATACA
Ces1d	ATGCGCCTCTACCCTCTGATA	AGCAAATCTCAAGGAGCCAAG
Dgat2	GCGCTACTTCCGAGACTACTT	GGGCCTTATGCCAGGAAACT
Pcyt1a	GATGCACAGAGTTCAGCTAAAGT	TGGCTGCCGTAAACCAACTG
Pcyt2	TGTGTTCACGGCAATGACATC	TTCCCGGTACTCAGAGGACAT
Pemt	TTGGGGATTCGTGTTTGTGCT	CACGCTGAAGGGAAATGTGG
Ptdss1	GCAGGACTCTGAGCAAGGATG	GCAGGACTCTGAGCAAGGATG
Scd1	TTCTTGCGATACACTCTGGTGC	CGGGATTGAATGTTCTTGTCGT
Fabp4	TGGAATGTGTTATGAAAGGC	AATTGCTTGCTTATTAGTGGAA
Fabp5	TCTCTGGTATTCTTCAGGATC	TCATTCGATAGGAATAGTCTCAA
Rbp4	GAAGTTTGAAGATTTCTGATTAGC	TAGGAAGATGGTGACTATATGTT
Retn	GGTCTGGAAATGAAGAATGAG	GGAGTGATGGACAAGTAGTAA
Lep	TACAGGTTGACTATCCCTTATC	GCAGTGTCTATACGGTATTATG
Adipo	TTGAGAGTCCTGAGTATTATCC	AGTCATGCGAATATTGTGAAG
Il-1a	GTGTTGCTGAAGGAGTTG	ATCTGGAAGTCTGTCATAGAG
Il-1b	GCAACTGTTCCTGAACTCAACT	ATCTTTTGGGGTCCGTCAACT
Il-2	TGAGCAGGATGGAGAATTACAGG	GTCCAAGTTCATCTTCTAGGCAC
Il-4	GGTCTCAACCCCCAGCTAGT	GCCGATGATCTCTCTCAAGTGAT
Il-6	CTACGAAGAACTGACAATATGAA	GAGGTAAACTTATACATTCCAAGA
Il-12a	CAATCACGCTACCTCCTCTTTT	CAGCAGTGCAGGAATAATGTTTC
Il-12b	TGGTTTGCCATCGTTTTGCTG	ACAGGTGAGGTTCACTGTTTCT
Ifng	ATGAACGCTACACACTGCATC	CCATCCTTTTGCCAGTTCCTC
Tnfa	CCCTCACACTCAGATCATCTTCT	GCTACGACGTGGGCTACAG
